# DrEFO: A Fermented Oil Rich in Dihydroquercetin With High Antioxidant Activity

**DOI:** 10.1111/jocd.70612

**Published:** 2025-12-18

**Authors:** Xing Meng, Yidan Sun, Xiaojie Li, Yanyong Xu, Guimei Xie, Song Jiao, Yuele Lu

**Affiliations:** ^1^ Beijing Maosi Business&Trade Co., Ltd Beijing China; ^2^ Hangzhou Jajale Biotech Co., Ltd Hangzhou China; ^3^ College of Biotechnology and Bioengineering Zhejiang University of Technology Hangzhou China

**Keywords:** cell culture, dihydroquercetin rich oil, formulation/stability, skin barrier, skincare efficacy

## Abstract

**Objective:**

The study aims to tackle the limitation of natural plant‐based oils in insufficient potency for addressing complex skin issues and explore a strategy to enhance the value of natural oils in skincare. A dihydroquercetin (DHQ) enriched eleven‐seed fermented oil (DrEFO) was achieved by fermenting an eleven‐seed oil (ESO).

**Materials and Methods:**

The distinctive antioxidant property of DHQ was first measured by DPPH radical scavenging experiment to evaluate its long‐term antioxidant capacity and its continuous antioxidant effect. To assess the distinctive long‐term antioxidation property of DHQ, DHQ was subjected to harsh real‐life conditions such as UV stress and thermal attack and monitored its antioxidant activity. To determine its continuous antioxidant effect, DPPH was continuously introduced into the system and the antioxidation result was measured. Then, a *S. cerevisiae* strain was engineered via synthetic biology approaches to produce DHQ, which was then used for large‐scale fermentation of DrEFO. The antioxidant property of DrEFO was tested by measuring the DPPH radical scavenging rate, as well as scavenging rates against ABTS, hydroxyl, superoxide anion, PTIO, and ROS radicals. The collagen synthesis boosting effect was also evaluated in cell models.

**Results:**

When exposed to harsh daily‐life conditions like UV stress and thermal attack, DHQ impressively retained its ability to combat oxidative stress over an extended period, clearly showcasing its long‐term protective effect. More significantly, DHQ was able to continuously neutralize DPPH radicals as they were introduced into the system. Thorough fermentation, more than 1000 ppm of DHQ was detected in DrEFO. DrEFO also showed outstanding antioxidant properties. Its DPPH radical scavenging rate was 4.3 times that of ESO, and it had high scavenging rates against multiple other radicals. In cell models, DrEFO significantly boosted collagen synthesis.

**Conclusion:**

These findings highlight DrEFO's potential as an effective skincare solution for various skin concerns. Moreover, this research pioneers a new way to enhance the value of natural oils in skincare by leveraging the power of fermentation. It transforms the capabilities of traditional natural oils to meet the diverse needs of consumers.

## 1 Introduction

In recent years, the beauty and skincare industry has experienced a paradigm shift. Consumers have become more astute and particular when seeking products that can truly improve and maintain the health and appearance of their skin. The demand for natural and effective skincare products has been rising rapidly [1, 2]. Consumers are no longer content with superficial remedies; instead, they increasingly prefer formulations that not only enhance the aesthetic appearance of the skin but also provide long‐term, sustainable health benefits [3]. Against this backdrop of the industry, the concept of “nourishing the skin with oil” has emerged and gradually gained popularity [2, 4, 5].

Consumers have come to realize that certain oils contain a rich array of beneficial components, such as essential fatty acids, antioxidants, and vitamins [4, 5, 6]. These components are crucial for maintaining the skin's natural barrier function [7, 8] and can deeply moisturize the skin, improve skin texture, and even out skin tone [9, 10, 11]. The importance of “nourishing the skin with oil” also lies in its versatility. It can be incorporated into various skincare routines, whether as a standalone moisturizer, a supplement to existing creams and lotions, or an ingredient in facial massages and treatments, and is suitable for different skin types such as dry, oily, and combination skin [4, 12, 13]. However, as the pursuit of more advanced and efficacious skincare solutions continues, the incorporation of compounds possessing potent skin care actives into oils has emerged as a remarkable and significant innovation.

Dihydroquercetin (DHQ), a flavonoid with a remarkable array of potent biological activities, has been the subject of extensive scientific research [14, 15, 16]. Its antioxidant properties are of utmost significance, as it can effectively scavenge free radicals, which are the root cause of many skin problems [16, 17, 18]. These free radicals, generated by environmental factors such as UV radiation, pollution, and stress, can wreak havoc on skin cells, leading to premature aging, wrinkles, and a loss of elasticity. By neutralizing these harmful agents, DHQ helps protect skin cells from oxidative stress‐induced damage, thereby playing a crucial role in maintaining the integrity and vitality of the skin. Furthermore, DHQ has demonstrated significant anti‐inflammatory capabilities [19, 20]. Inflammation is a common underlying factor in various skin conditions, including acne, rosacea, and eczema. By modulating the inflammatory response, this flavonoid can help soothe irritated skin, reduce redness and swelling, and promote a more even skin tone. Additionally, it has been found to stimulate collagen synthesis, a key protein that provides structural support to the skin [21]. As we age, collagen production naturally declines, resulting in sagging skin and the formation of wrinkles. The ability of DHQ to boost collagen synthesis offers the potential to reverse or at least slow down these age‐related changes, improving skin elasticity and firmness. Thus, the integration of DHQ into oil holds the promise of bestowing a multitude of advantages upon the skin.

However, a significant challenge lies in the fact that DHQ is virtually insoluble in oils. This insolubility means that a straightforward mixing process cannot be employed to incorporate DHQ into oils. The lack of solubility prevents the uniform distribution of DHQ within the oil matrix, making it difficult to harness its full potential for skincare products. To tackle this challenge, fermented oils have emerged as an innovative solution. Their microbial‐fermented structure, rich in polar derivatives, free fatty acids, and biosurfactant compounds [22, 23], would significantly improve DHQ solubility.

In this study, we sought to develop a DHQ‐enriched eleven‐seed fermented oil (DrEFO). Through genetic engineering of microorganisms, DHQ was bio‐synthesized and enriched in the oil matrix during fermentation of eleven‐seed oil (ESO), thereby generating DrEFO. Our initial step involved measuring and comparing the long‐term antioxidant capacity and sustained radical scavenging effect of DHQ against conventional antioxidants—Vitamin C (VC), Vitamin E (VE), and ergothioneine (EGT) to characterize DHQ's unique antioxidant profile. Subsequently, a microbial strain was engineered for DHQ production and applied to ferment ESO, yielding DrEFO. Finally, the bioactive properties of DrEFO were validated in skincare applications. A series of in vitro assays were conducted to evaluate its antioxidant and collagen I promotion capabilities.

## 2 Materials and Methods

### 2.1 Materials

All materials used in this study were commercially available, with detailed information provided in the Supporting Information.

### 2.2 Tolerance Evaluation of *Escherichia coli* , *Bacillus subtilis* , and *Saccharomyces cerevisiae* to DHQ

*E. coli and B. subtilis* seed cultures were inoculated into LB media and cultured at 37°C for 12 h, which were then transferred to LB media containing 1 g/L DHQ and cultured at 37°C for 6 h. LB media without DHQ was used as a control.

Similarly, *S. cerevisiae* was inoculated into YPD media and cultured at 30°C for 24 h, which was then transferred to YPD media containing 1 g/L DHQ and cultured at 30°C for 12 h. YPD media without DHQ was used as a control.

After incubation, determine the viable cell counts by the plate counting method.

### 2.3 Screening of DHQ Tolerant Microorganism

The soil sample was collected from the root zone of larch trees in the Mohe area, Daxing'anling, China. The sample was first cultured in enrichment media at 30°C overnight, followed by 10^4^ and 10^5^ fold dilutions, which were then screened on YPD solid media containing 5 g/L of DHQ. The enrichment media was composed of 2 g glucose, 0.2 g yeast extract, 0.2 g NH_4_NO_3_, 0.3 g KH_2_PO_4_, 0.5 g Na_2_HPO_4_·12H_2_O, and 0.05 g MgSO_4_·7H_2_O per liter. The YPD solid media was composed of 10 g yeast extract, 20 g tryptone, 20 g glucose, and 20 g agar per liter.

### 2.4 Construction of DHQ Producing Strain

In order to achieve DHQ production, heterologous genes *F3h* from *Solanum lycopersicum* encoding flavonoid‐3‐hydroxylase (F3H) and F3′h from *Gerbera hybrid* encoding flavonoid‐3′‐hydroxylase (F3′H) were integrated into chromosomes of *S. cerevisiae*. The endogenous genes, promoter, and terminator sequences were cloned from the genomic DNA of *S. cerevisiae* CEN.PK2‐1D, and the synthesized heterologous genes were provided by Tsingke Biotech Co. Ltd. (Beijing, China) as cloning templates. The CRISPR/Cas9 system was employed to integrate DNA into the yeast genome [24], and the specific guide RNAs were constructed using the online website (http://www.rgenome.net/cas‐designer/). The DNA fragments were transformed into *S. cerevisiae* cells by using the lithium acetate method [24]. The DNA fragments were assembled using a one‐step cloning kit (Vazyme Biotech Co. Ltd., Nanjing, China). In addition, the endogenous NADPH regeneration genes (Tyr1, Gnd1, and Zwf1) were overexpressed to increase NADPH flux. The final DHQ‐producing strain was named *S. cerevisiae* Sc04.

### 2.5 Fermentation Production of DrEFO

*S. cerevisiae* Sc04 was cultured in the YPD media for 12 h at 30°C to obtain *S. cerevisiae* seed. *Lactobacillus* [23] was cultured in the PSB seed media for 12 h at 30°C to obtain *Lactobacillus* seed. The PSB seed media was composed of 7 g tryptone, 5 g beef extract, 4 g yeast extract, 2 g glucose, 2 g K_2_HPO_4_, 4 g sodium acetate, 2 g Ammonium Citrate Tribasic, and 0.2 g MgSO_4_ per liter. The *S. cerevisiae* seed was then inoculated into the yeast fermentation media at an inoculation rate of 5% and cultured for 24 h at 30°C, and then 5 g/L of naringenin (NAR) was added and cultured for another 24 h to obtain the *S. cerevisiae* pre‐fermentation liquid. The yeast fermentation media was composed of 18 g rice powder, 20 g soybean extract, 10 g glucose per liter. The pH was set to 6.0 and automatically controlled by feeding ammonia solution (28%, v/v). Air flow was constantly set at 1 vvm, and the dissolved oxygen concentration was set at 30% of air saturation by automatically controlling the agitation speed (300–1000 rpm). When the initial glucose was depleted, the feeding solution (5 g MgSO_4_·7H_2_O, 700 g glucose per liter) was automatically fed to the bioreactor at a feeding rate of 2 g/(L·h). The *Lactobacillus* seed was inoculated into the *Lactobacillus* fermentation media at an inoculation rate of 5% and cultured for 8 h at 30°C, and then ESO was added and cultured for another 6 h to obtain the *Lactobacillus* pre‐fermentation liquid. The *Lactobacillus* fermentation media was composed of 18 g glucose, 20 g soybean extract, 10 g corn powder, and 20 g whey powder per liter. The initial pH was set to 6.0 and automatically controlled by feeding 1% NaOH solution. Air flow was constantly set at 0.1 vvm, and the agitation speed was constantly set at 200 rpm. The *S. cerevisiae* pre‐fermentation liquid and *Lactobacillus* pre‐fermentation liquid were mixed and cultured for 12 h at 25°C. The pH was set at 6.0 and automatically controlled by feeding 1% NaOH solution. Air flow was constantly set at 0.5 vvm, and the agitation speed was constantly set at 400 rpm. The feeding solution (5 g MgSO_4_·7H_2_O, 700 g glucose per liter) was automatically fed to the bioreactor at a feeding rate of 2 g/(L·h). The oil phase was collected and extracted to obtain DrEFO.

### 2.6 DPPH Radical Scavenging Rate Assay of DHQ, VC, VE, and EGT

20 μL of sample solutions containing 300 μM of DHQ, VC, VE, and EGT respectively was each mixed with 180 μL of DPPH solution. These mixtures were then incubated at room temperature for 30 min. After incubation, the absorbance of each mixture was measured at a wavelength of 517 nm using a spectrophotometer and recorded as T1 for each respective sample.

As a reference, for each antioxidant, the DPPH solution was replaced with ethanol and mixed with 20 μL of the sample solution of the corresponding antioxidant (DHQ, VC, VE, or EGT). The same incubation and measurement procedures were followed, and the absorbance was recorded as T0 for each case.

As a control, 20 μL of water was mixed with 180 μL of DPPH solution and incubated at room temperature for 30 min. The absorbance at the wavelength of 517 nm was measured and recorded as C1. As a reference for the control, the DPPH solution was replaced with ethanol and mixed with 20 μL of water. The same procedure was performed, and the absorbance was recorded as C0.

The DPPH radical scavenging rate for each of DHQ, VC, VE, and EGT was calculated using the formula: [(C1 − C0) − (T1 − T0)]/(C1 − C0) × 100%. This allowed for a direct comparison of the antioxidant capabilities of DHQ, VC, VE, and EGT in terms of their ability to scavenge DPPH radicals.

### 2.7 Measurement of the Long‐Term Antioxidation Property of DHQ, VC, VE, and EGT

To comprehensively evaluate the distinctive long‐term antioxidation property of DHQ, it was exposed to harsh real‐life conditions, specifically UV stress and thermal attack, while closely monitoring its antioxidant activity.

For assessing the thermal stability of DHQ, VC, VE, and EGT, solutions containing 300 μM of each compound were incubated at 60°C. The DPPH radical scavenging rates were then measured at 0, 2, 16, 24, 40, 48, 64, 72, 88, and 96 h.

Regarding the evaluation of their UV stress stability, 300 μM solutions of DHQ, VC, VE, and EGT were exposed to UV light. The DPPH radical scavenging rates were determined at 0 min, 20 min, 40 min, 60 min, and 120 min.

### 2.8 Measurement of the Continuous Antioxidation Property of DHQ, VC, VE, and EGT

To comparatively assess the continuous antioxidant efficacy of DHQ, VC, VE, and EGT, a series of experiments were designed. Initially, 20 μL of 300 μM DHQ, VC, VE, and EGT sample solutions were respectively incubated with 180 μL of DPPH solution. In addition to the initial addition of DPPH at 0 h, supplementary DPPH was introduced into the reaction systems at 2, 4, 6, and 8 h. The DPPH radical scavenging rates were then measured and recorded at 2, 4, 6, 8, 10, 12, and 24 h.

### 2.9 DHQ Content Measurement of DrEFO

DHQ content in DrEFO was determined according to the reference with modifications [25]. Briefly, DHQ was first extracted from DrEFO with methanol. 2 mL of DrEFO was mixed vigorously with 2 mL of methanol, and the methanol phase was collected for High‐Performance Liquid Chromatography (HPLC) analysis. The analysis was performed on a HiQ sil‐C18 column, and the mobile phase was acetonitrile‐water‐acetic acid (18:82:0.1, v/v/v) with a 1.0 mL/min flow rate. The absorbance was measured at a wavelength of 294 nm.

### 2.10 DPPH Radical Scavenging Rate Assay of DrEFO

20 μL of sample with different concentrations of DrEFO was mixed with 180 μL of DPPH solution and incubated at room temperature for 30 min. The absorbance was measured at a wavelength of 517 nm using the spectrophotometer after incubation and recorded as T1. As a reference, the DPPH solution was replaced with ethanol and mixed with 20 μL of sample with different concentrations of DrEFO. The same procedure was performed, and the absorbance was recorded as T0. As a control, 20 μL of water was mixed with 180 μL of DPPH solution and incubated at room temperature for 30 min. Measure the absorbance at the same wavelength and record it as C1. As a reference, the DPPH solution was replaced with ethanol and mixed with 20 μL of water. The same procedure was performed, and the absorbance was recorded as C0. The DPPH radical scavenging rate was calculated as [(C1‐C0)‐(T1‐T0)]/(C1‐C0)*100%.

### 2.11 ABTS Radical Scavenging Rate Assay of DrEFO

The ABTS radical scavenging rate assay was performed by using a Total Antioxidant Capacity Detection Kit according to the protocol. Briefly, 10 μL of sample with different concentrations of DrEFO was mixed with 200 μL of ABTS working solution and incubated at room temperature for 6 min. The absorbance was measured at a wavelength of 734 nm using the spectrophotometer after incubation and recorded as T1. As a reference, the ABTS working solution was replaced with PBS and mixed with 10 μL of sample with different concentrations of DrEFO. The same procedure was performed, and the absorbance was recorded as T0. As a control, 10 μL of water was mixed with 200 μL of ABTS working solution and incubated at room temperature for 6 min. Measure the absorbance at the same wavelength and record it as C1. As a reference, the ABTS working solution was replaced with PBS and mixed with 10 μL of water. The same procedure was performed, and the absorbance was recorded as C0. The ABTS radical scavenging rate was calculated as [(C1 − C0) − (T1 − T0)]/(C1 − C0) × 100%.

### 2.12 Hydroxyl Radical Scavenging Rate Assay of DrEFO

The hydroxyl radical scavenging rate assay was performed by using a Hydroxyl Radical Assay Kit according to the protocol. 0.2 mL of sample with different concentrations of DrEFO was mixed with 0.2 mL of substrate application solution and 0.4 mL of reagent III application solution. The mixture was incubated at 37°C for 1 min and 2 mL of chromogenic agent was added to stop the reaction. The absorbance was measured at a wavelength of 550 nm and recorded as T1. As a blank control, the DrEFO sample was replaced with water, and the absorbance was measured and recorded as C1. The hydroxyl radical scavenging rate was calculated as (C1 − T1)/C1 × 100%.

### 2.13 Superoxide Anion Radical Scavenging Rate Assay of DrEFO

The superoxide anion radical scavenging rate assay was performed by using a Superoxide Anion Radical Assay Kit according to the protocol. 0.05 mL of sample with different concentrations of DrEFO was mixed with 1 mL of reagent I, 0.1 mL of reagent II, 0.1 mL of reagent III, and 0.1 mL of reagent IV. The mixture was incubated at 37°C in a water bath for 40 min, and 2 mL of chromogenic agent was added, which was then transferred to room temperature. After 10 min, the absorbance of the mixture was measured at a wavelength of 550 nm and recorded as T1. As a blank control, the sample solution was replaced with water, and the absorbance was measured and recorded as C1. The superoxide anion radical scavenging rate was calculated as (C1 − T1)/C1 × 100%.

### 2.14 PTIO Radical Scavenging Rate Assay of DrEFO

20 μL of sample with different concentrations of DrEFO was mixed with 180 μL of PTIO solution and incubated at room temperature for 30 min. The absorbance was measured at a wavelength of 557 nm using the spectrophotometer after incubation and recorded as T1. As a reference, the PTIO solution was replaced with ethanol and mixed with 20 μL of sample with different concentrations of DrEFO. The same procedure was performed, and the absorbance was recorded as T0. As a control, 20 μL of water was mixed with 180 μL of PTIO solution and incubated at room temperature for 30 min. Measure the absorbance at the same wavelength and record it as C1. As a reference, the PTIO solution was replaced with ethanol and mixed with 20 μL of water. The same procedure was performed, and the absorbance was recorded as C0. The PTIO radical scavenging rate was calculated as [(C1 − C0) − (T1 − T0)]/(C1 − C0) × 100%.

### 2.15 ROS Radical Scavenging Rate Assay of DrEFO

The DCFH‐DA method was adopted to measure the ROS radical scavenging rate of DrEFO in HaCaT keratinocyte cells [26]. Cells were seeded into a 24‐well culture plate at a density of 1 × 10^5^ cells/well and cultured in a CO_2_ incubator at 37°C overnight. Then, the cells were exposed to UVB (300 mJ/cm^2^) and treated with 0.0125%, 0.025%, or 0.05% of DrEFO. After incubation for 24 h, the cells were treated with DCFH‐DA solution (10 μM) and incubated for another 30 min. Then, wash the cells with serum‐free DMEM culture medium four times and measure the fluorescence at an excitation wavelength of 488 nm.

### 2.16 Collagen I Promotion Assay of DrEFO

The human fibroblasts were inoculated into a 24‐well culture plate at a density of 8 × 10^4^ cells/well and cultured in a CO_2_ incubator at 37°C overnight. The cultured cells were then exposed to UVA radiation (9 J/cm^2^) and treated with 0.0125%, 0.025%, or 0.05% of DrEFO. After incubation for 24 h, the collagen I expression level was measured using the immunofluorescence detection method.

## 3 Results

### 3.1 Antioxidation Capacity Comparison of DHQ With VC, VE, and EGT

**FIGURE 1:**
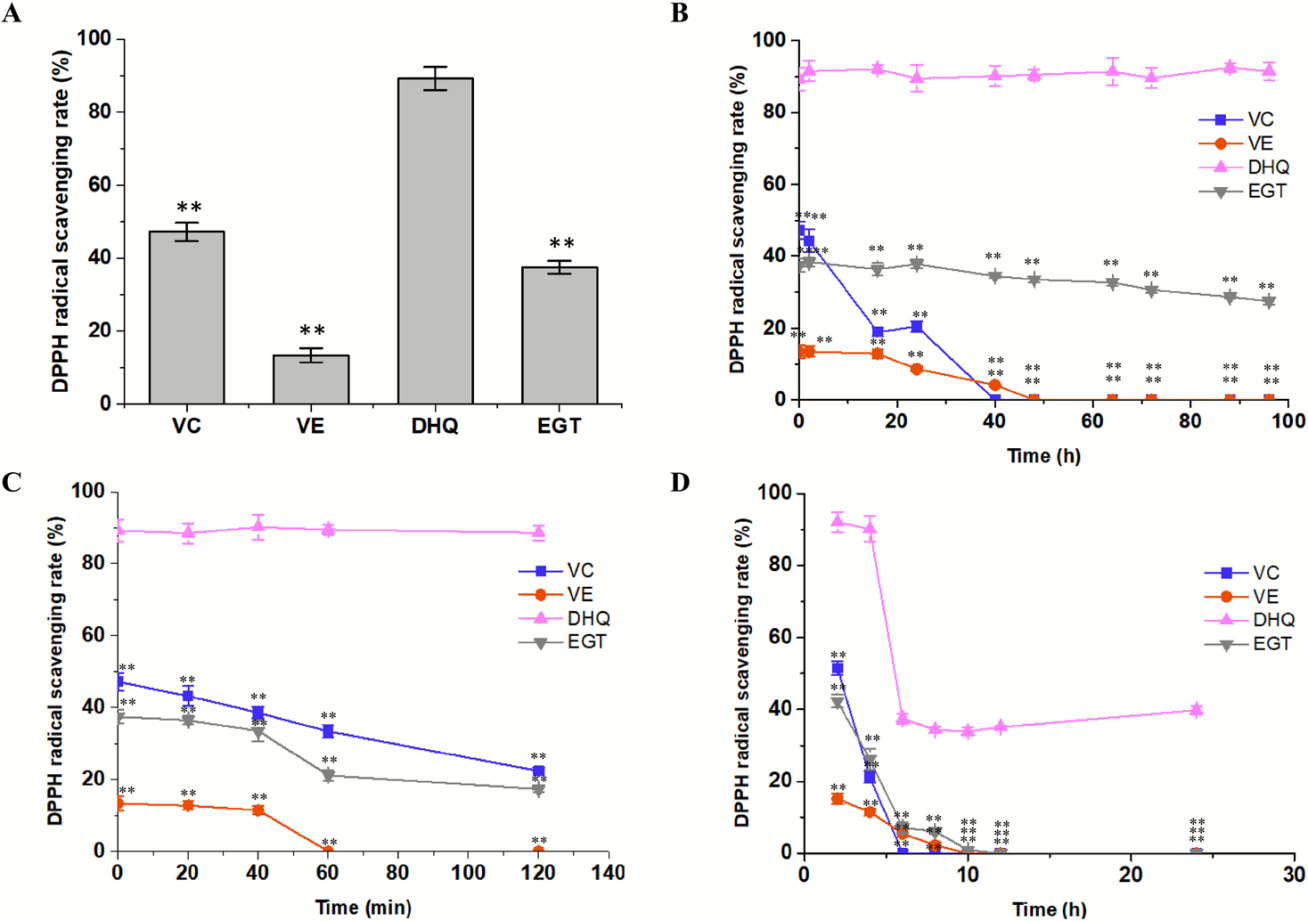
Antioxidation capacity evaluation of DHQ in comparison with VC, VE, and EGT. (A) DPPH radical scavenging rate of 30 μM of DHQ, VC, VE, and EGT. (B) Thermal stability evaluation of DHQ, VC, VE, and EGT at 60°C. (C) UV stability evaluation of DHQ, VC, VE, and EGT under UV exposure. (D) Continuous antioxidation property comparison of DHQ, VC, VE, and EGT. DPPH was supplemented at 2, 4, 6, and 8 h. *p* < 0.01 was indicated as **. Error bars represent standard deviation from biological triplicates.

VC, VE, and EGT are well‐established antioxidants renowned for their potent antioxidation capabilities. In this study, the antioxidation capacity of DHQ was compared with that of VC, VE, and EGT using the DPPH radical scavenging rate assay. As depicted in Figure 1A, when the final concentration of DHQ was 30 μM, its DPPH radical scavenging rate reached 89.2% ± 3.2%. This value was 1.89‐fold, 6.66‐fold, and 2.38‐fold higher than those of VC, VE, and EGT, respectively. These results demonstrate the remarkable antioxidation capacity of DHQ, highlighting its potential to outperform some of the commonly used antioxidants in scavenging DPPH radicals and thus protecting against oxidative stress.

### 3.2 Comparison of the Long‐Term Antioxidation Property of DHQ, VC, VE, and EGT

In our daily life, the skin is constantly exposed to a variety of harsh environmental factors, prominently including UV stress and thermal attack. The stability of antioxidants under such conditions is a crucial determinant of their overall antioxidation property. To investigate this aspect, in this study, DHQ, VC, VE, and EGT were subjected to either a 60°C thermal environment or UV light exposure, followed by the measurement of their DPPH radical scavenging rates.

As illustrated in Figure 1B, when 30 μM of DHQ was incubated at 60°C for 96 h, its DPPH radical scavenging rate remained at 91.5% ± 2.4%. In contrast, the DPPH radical scavenging rate of EGT under the same conditions dropped to 27.5% ± 0.8%. Notably, VC completely lost its antioxidation capacity after 40 h of incubation at 60°C, and VE followed suit after 48 h.

Figure 1C further reveals that after 120 min of UV exposure, the radical scavenging rate of DHQ remained as high as 88.6% ± 2.1%. In comparison, the rates for VC, EGT, and VE were merely 22.4%, 17.3%, and 0%, respectively. These results unequivocally demonstrate that DHQ exhibits superior stability compared to VC, VE, and EGT, particularly when confronted with UV radiation and thermal exposure. This enhanced stability positions DHQ as a more reliable antioxidant candidate in real‐world scenarios where the skin is frequently challenged by these harsh conditions.

### 3.3 Comparison of the Continuous Antioxidation Property of DHQ, VC, VE, and EGT

In our daily life, free radicals are constantly generated by our internal cellular respiration and external factors like pollution and UV radiation. Consequently, the availability of antioxidants capable of offering continuous protection against these free radicals becomes of paramount significance.

In this study, a comprehensive comparison was conducted on the continuous antioxidation properties of DHQ, VC, VE, and EGT. As depicted in Figure 1D, DHQ demonstrated remarkable resilience, maintaining a high level of radical‐scavenging activity even after a 24 h period. This indicates its outstanding ability to continuously neutralize free radicals over an extended time frame. Conversely, VC, VE, and EGT experienced a drastic decline in their antioxidant efficacy. By the end of the 24 h assessment, they had almost entirely lost their capacity to scavenge free radicals. This stark contrast in performance clearly highlights the unique and persistent nature of DHQ's continuous antioxidation capabilities.

### 3.4 Production of DHQ by Engineered *S. cerevisiae* Sc04

Having demonstrated the outstanding antioxidation capacity of DHQ, our next focus was to produce DHQ‐enriched DrEFO during fermentation. This necessitated the engineering of a DHQ‐producing microbial host. DHQ is known to exhibit potent antimicrobial effects against microorganisms such as *Staphylococcus aureus* , *B. subtilis* , *E. coli* , etc., a finding replicated in our study (Figure S1). To enable high‐titer DHQ production, we first screened for DHQ‐resistant strains and isolated one capable of growing on media supplemented with 5 g/L of DHQ. ITS region sequencing and phylogenetic analysis confirmed its classification within the *S. cerevisiae* family.

**FIGURE 2:**
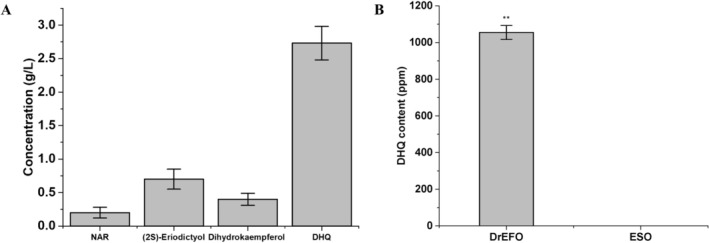
DHQ measurement result. (A) DHQ titer of the fermentation by *S. cerevisiae* Sc04. (B) DHQ content measurement of DrEFO. *p* < 0.01 was indicated as **. Error bars represent standard deviation from biological triplicates.

DHQ biosynthesis from NAR involves two enzymatic steps catalyzed by F3'H and F3H, generating (2*S*)‐eriodictyol and dihydrokaempferol as intermediates. Thus, the *F3h* gene from *Solanum lycopersicum* encoding F3H and *the F3'h* gene from *Gerbera hybrid* encoding F3'H were overexpressed in *S. cerevisiae*. Cytochrome P450 reductase (CPR), the redox partner of F3'H, was co‐overexpressed to enhance catalytic efficiency. The resulting strain was used for DHQ production. As shown in Figure 2A, with the addition of 5 g/L of NAR as substrate, 2.73 g/L of DHQ was finally obtained.

### 3.5 Production of DrEFO

In a previous study, a fermented oil was synthesized, generating polar derivatives, free fatty acids, and biosurfactant compounds. These components endowed the oil with enhanced emulsifying activity and reduced surface tension, potentially improving the solubility of DHQ in the oil matrix [23]. Consequently, the production method was integrated with the fermentation of engineered *S. cerevisiae* , yielding DrEFO. The DHQ content of DrEFO was measured using the HPLC method by first extracting DHQ from DrEFO with methanol. As depicted in Figure 2B and Figure S2, the DHQ content in DrEFO was 1055 ± 38 ppm. This indicates a relatively consistent presence of DHQ within this particular sample. In contrast, when the same method was applied to analyze ESO, DHQ was not detectable. These results highlight the significant difference in the DHQ composition between DrEFO and ESO, which may have implications for their respective biological activities and potential applications.

### 3.6 Antioxidant Measurement of DrEFO

**FIGURE 3:**
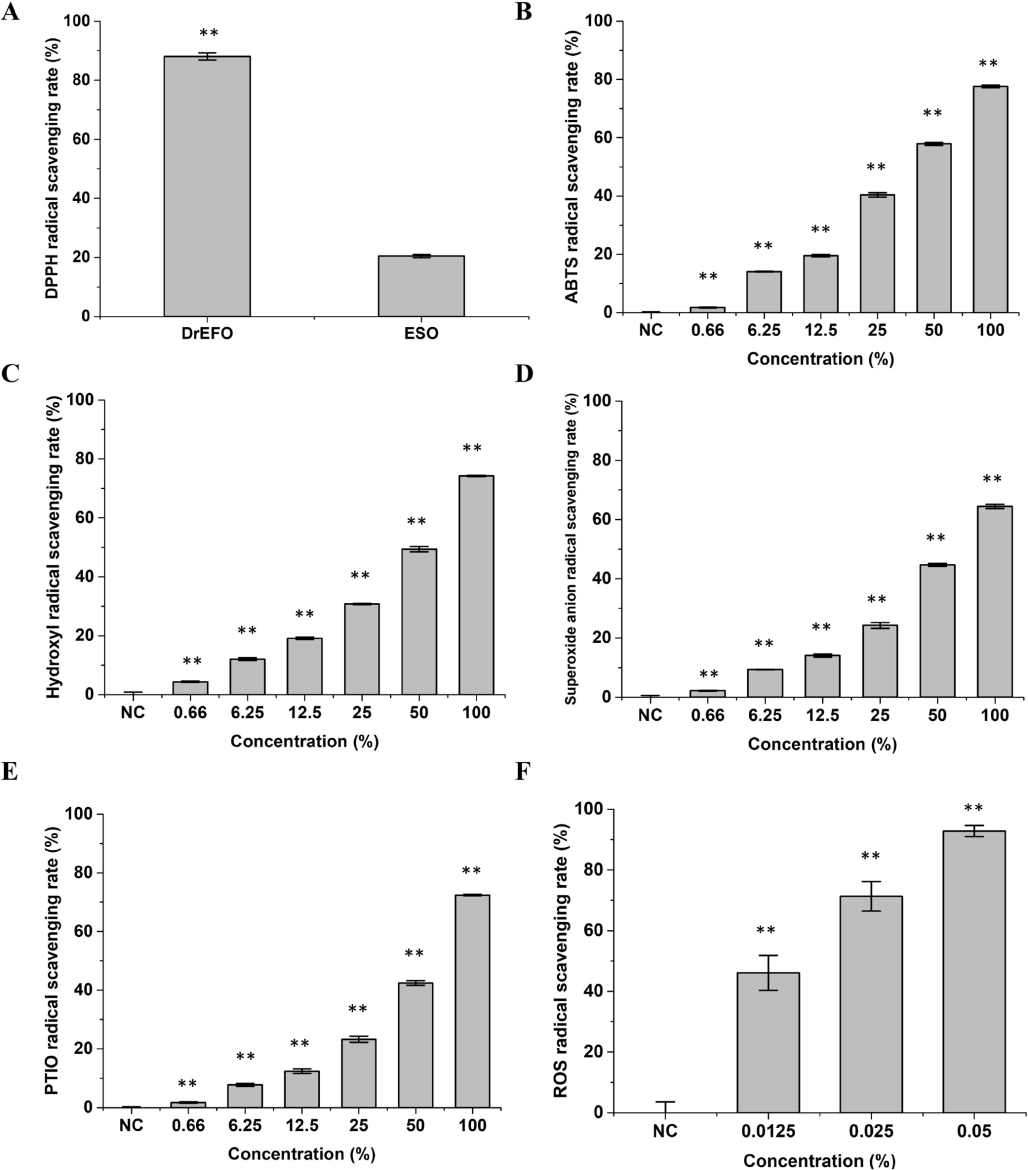
Antioxidant activity measurement of DrEFO. (A) DPPH radical scavenging rate comparison of DrEFO and ESO. (B–F) Scavenging rate of DrEFO with different concentrations towards ABTS radicals (B), hydroxyl radicals (C), superoxide anion radicals (D), PTIO radicals (E), and ROS radicals (F). NC, no DrEFO addition. *p* < 0.01 was indicated as **. Error bars represent standard deviation from biological triplicates.

Since DHQ is a super antioxidant, the antioxidant capabilities of DrEFO and ESO were compared by measuring their DPPH radical scavenging rates. As presented in Figure 3A, the DPPH radical scavenging rate of DrEFO reached 88.1% ± 3.2%, which was 4.3‐fold that of ESO, clearly demonstrating that DrEFO possesses outstanding antioxidant properties.

Besides DPPH radicals, ABTS radicals, hydroxyl radicals, superoxide anion radicals, PTIO radicals, and ROS radicals are common radicals found in biological systems. These radicals can be generated during normal metabolic processes, such as cellular respiration, or in response to external stressors like radiation, toxins, and inflammation. Their presence often leads to oxidative stress, which can damage cellular components including DNA, proteins, and lipids, potentially contributing to various diseases and aging‐related processes. Thus, to comprehensively evaluate the antioxidant capacity of DrEFO, its scavenging abilities towards these common radicals were measured. The results are shown in Figure 3B,E. Impressively, DrEFO demonstrated high radical scavenging rates against all types of radicals tested. Specifically, 100% pure DrEFO exhibited scavenging rates of 77.60% ± 0.42% for ABTS radicals, 74.22% ± 0.21% for hydroxyl radicals, 64.42% ± 0.73% for superoxide anion radicals, and 72.44% ± 0.25% for PTIO radicals. The determination of the effect of DHQ on ROS was carried out through the human immortalized keratinocyte cell model. As shown in Figure 3F and Supplemental Figure S1, an astonishing finding was that as little as 0.05% of DrEFO was capable of neutralizing 92.8% ± 1.8% of ROS radicals. This remarkable result further emphasizes the potent antioxidant potential of DrEFO, not only in in vitro chemical assays but also in a more physiologically relevant cell‐based model.

### 3.7 Collagen I Promotion Activity of DrEFO by Cell‐Based Model

**FIGURE 4:**
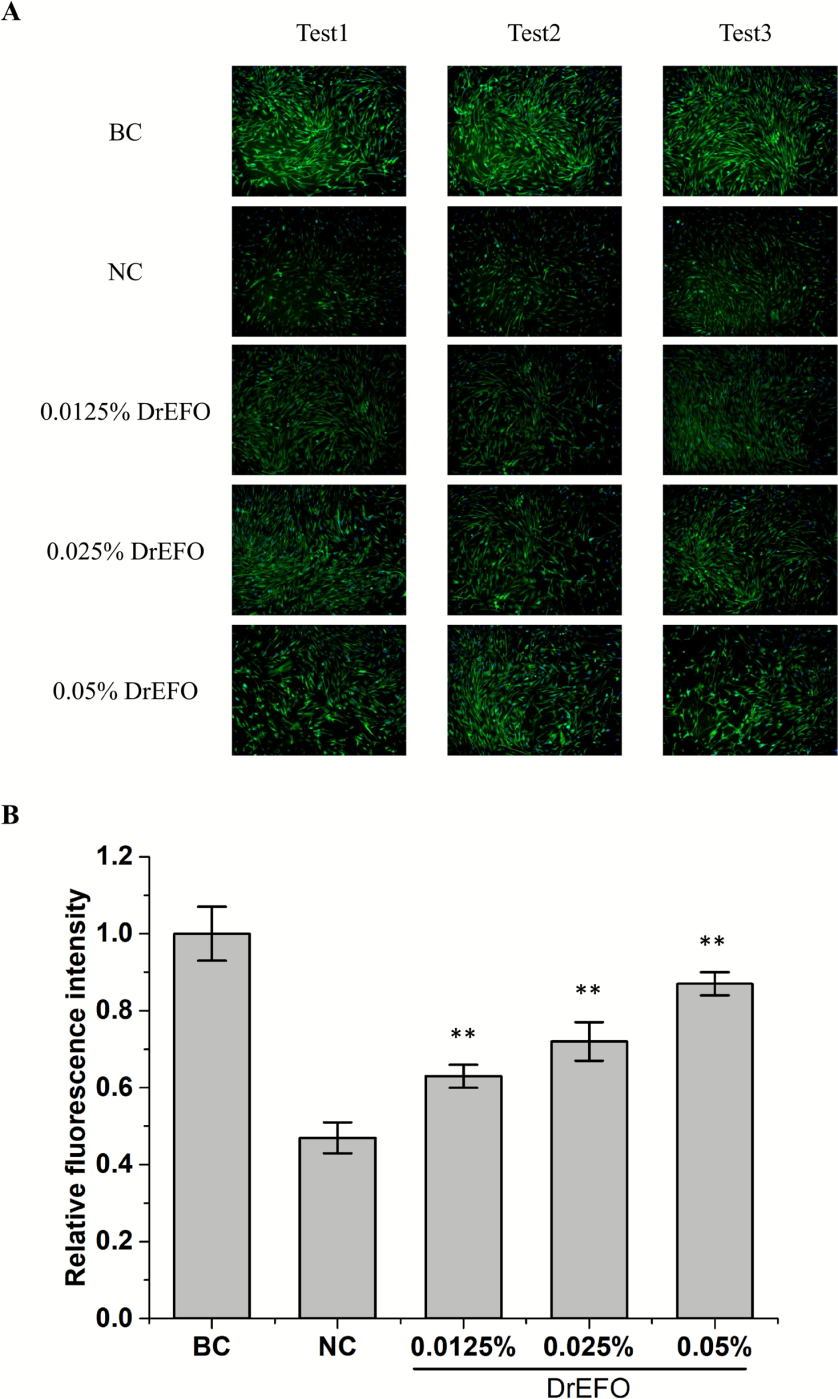
Effects of DrEFO on collagen production in human fibroblast through immunofluorescence assay. (A) Summary diagram of immunofluorescence staining results for collagen I. (B) Relative fluorescence intensity of collagen I. *p* < 0.01 was indicated as **. Error bars represent standard deviation from biological triplicates.

Collagen, a fibrous protein in the skin's dermis, is vital for filling the spaces between cells and preserving tissue flexibility. This makes effectively increasing skin collagen levels a key objective in cosmetic research. To assess the collagen I promotion activity of DrEFO, human fibroblasts were utilized. Immunofluorescence technology was employed to detect changes in the expression level of collagen I. Figure 4 shows that, when compared to the UVA‐radiation sample (NC), the relative expression of collagen I increased significantly. When the cells were treated with 0.0125%, 0.025%, and 0.05% of DrEFO, the relative expression of collagen I rose from 0.47 to 0.63, 0.72, and 0.87, respectively. This indicates that DrEFO can significantly boost collagen synthesis.

## 4 Discussion

The present study successfully developed a novel DHQ‐enriched fermented oil by integrating microbial fermentation with genetic engineering. Our findings demonstrate that this approach not only overcame the inherent solubility limitations of DHQ in oils but also yielded a formulation with superior and sustained antioxidant activity and collagen‐promoting effects, positioning DrEFO as a promising ingredient for advanced skincare applications.

The exceptional antioxidant capacity of DHQ, as evidenced by its significantly higher DPPH radical scavenging rate compared to VC, VE, and EGT, underscores its potential as a potent antioxidant agent. More importantly, DHQ exhibited remarkable stability under prolonged thermal stress and UV exposure conditions highly relevant to real‐world skincare scenarios where products are often subjected to similar environmental challenges. This stability is a critical advantage over conventional antioxidants like VC and VE, which rapidly lose activity under the same conditions. The ability of DHQ to maintain high radical‐scavenging activity over 24 h further highlights its potential for providing prolonged protection against continuous free radical generation, a common phenomenon in skin metabolism and under environmental stressors.

A key innovation of this study lies in the strategic use of fermentation to address the challenge of incorporating the flavonoid DHQ into an oil matrix. By engineering a DHQ‐resistant *S. cerevisiae* strain capable of biosynthesizing DHQ during the fermentation of ESO, we enabled the in situ production and enrichment of DHQ within the oil. The resulting DrEFO contained a substantial concentration of DHQ, which was undetectable in the non‐fermented control oil (ESO). This suggests that the fermentation process, potentially through the generation of polar derivatives, free fatty acids, and biosurfactants (Figure S3), created a microenvironment conducive to the integration and stabilization of DHQ within the oil matrix [23]. This method presents a significant advancement over direct mixing, which is ineffective due to the insolubility of crystalline flavonoids like DHQ in oils.

DrEFO exhibits a significantly enhanced antioxidant profile, as evidenced by its potent scavenging activity against a broad spectrum of radicals—an effect directly attributed to the successful incorporation of DHQ. Additionally, DrEFO stimulates collagen I synthesis in human fibroblasts in a concentration‐dependent manner. While these in vitro findings are highly promising, it is important to acknowledge that the current study has limitations. The assessment of skin penetration, validation of efficacy in a living organism (in vivo), and a complete safety profile for DrEFO have not yet been fully established. Future work should therefore prioritize advanced skin penetration assays, comprehensive in vivo studies to confirm the anti‐aging and antioxidant benefits, and a thorough safety assessment to ensure both topical safety and bio‐compatibility, which are crucial steps for its development as a credible cosmetic or dermatological ingredient.

In conclusion, our study demonstrates that the fusion of genetic engineering and fermentation technology offers a feasible solution for enriching oils with bioactive compounds like DHQ. This research not only validates DrEFO as a highly promising candidate for innovative skincare products but also establishes a versatile biotechnological platform for developing next‐generation, efficacy‐driven fermented oil ingredients.

## Author Contributions

Conceptualization, X.M. and S.J.; methodology, Y.S., X.L., Y.L., and S.J.; investigation, X.M., Y.S., and X.L.; resources, X.M., X.L., Y.X., G.X., S.J., and Y.L.; data curation, Y.S. and S.J.; writing – original draft preparation, Y.S., X.L., Y.X., and G.X.; writing – review and editing, S.J. and Y.L. All authors have read and agreed to the published version of the manuscript.

## Conflicts of Interest

Authors declare that they have financial conflicts of interest as the production method of DrEFO has been covered by patent CN2025101497819 registered.

## Supporting information


**Data S1:** jocd70612‐sup‐0001‐supinfo.docx.

## Data Availability

The data that support the findings of this study are available from the corresponding author upon reasonable request.
