# Supplementary material for: DrEFO: A Fermented Oil Rich in Dihydroquercetin With High Antioxidant Activity
Source: J Cosmet Dermatol. 2025 Dec 18;24(12):e70612. doi: 10.1111/jocd.70612 (PMC12715063; doi:10.1111/jocd.70612)
Supplement: Supplementary file 1 — Data S1: jocd70612‐sup‐0001‐supinfo.docx. [file JOCD-24-e70612-s001.docx]

**Materials**

The materials utilized in this study included high-glucose Dulbecco's Modified Eagle Medium (DMEM) culture medium and Phosphate Buffered Saline (PBS) (VivaCell, Shanghai, China), (VivaCell, Shanghai, China), Fetal Bovine Serum (FBS) and tryptone (Gibco, Shanghai, China), MTT and DPPH (Sigma, Shanghai, China), DMSO (SINOPHARM, Beijing, China), Total Antioxidant Capacity Detection Kit and Reactive Oxygen Species (ROS) Detection Kit (Beyotime, Shanghai, China), Hydroxyl Radical Assay Kit and Superoxide Anion Radical Assay Kit-Colorimetric Method (Jiancheng, Nanjing, China), PTIO (Macklin, Shanghai, China), Human Collagen Type I (col-I) ELISA Kit (CUSABIO), primary antibody and secondary antibody (Abcam, Shanghai, China), ESO (Jajale Biotech, Hangzhou, China).


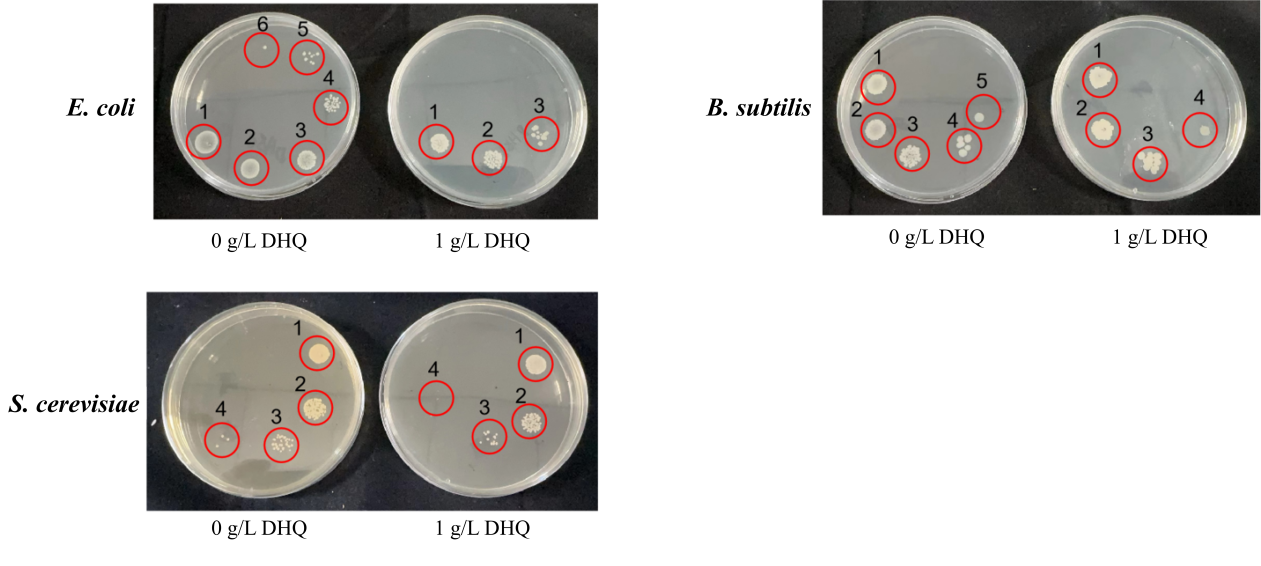


Supplementary Figure S1 Tolerance evaluation of *E. coli*, *B. subtilis* and *S. cerevisiae* to DHQ. The same numbers indicate that the concentrations of bacterial solutions used in the 0 g/L DHQ group and the 1 g/L DHQ group are identical.


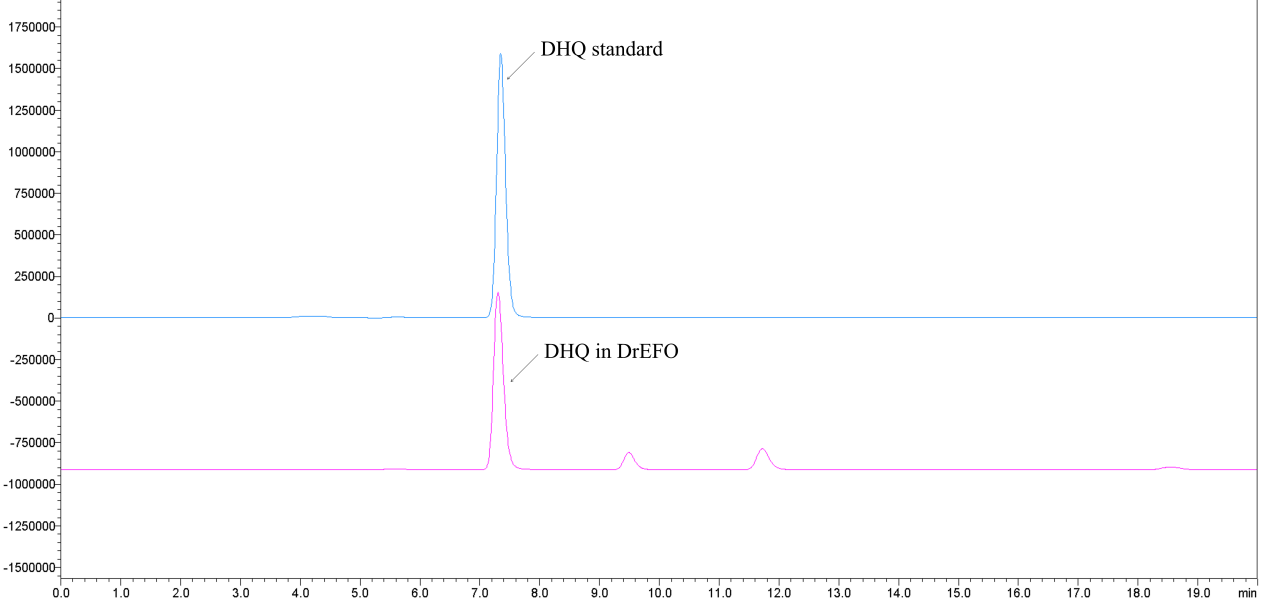


Supplementary Figure S2 HPLC identification of DHQ in DrEFO.


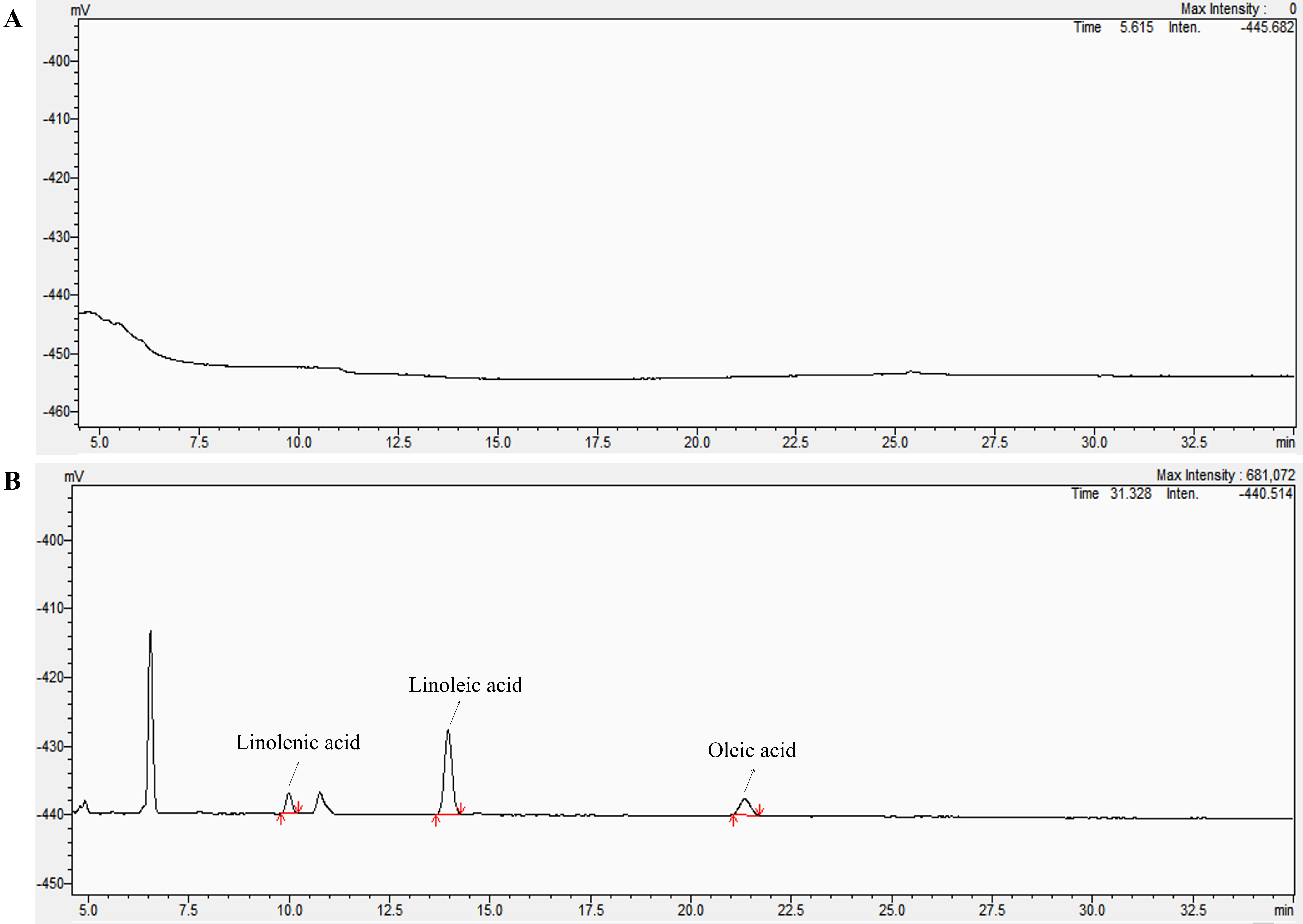


Supplementary Figure S3 Free fatty acid comparison of ESO (A) and DrEFO (B).
